# Discovering the mechanism of action of drugs with a sparse explainable network

**DOI:** 10.1016/j.ebiom.2023.104767

**Published:** 2023-08-24

**Authors:** Katyna Sada Del Real, Angel Rubio

**Affiliations:** a Departamento de Ingeniería Biomédica y Ciencias, TECNUN, Universidad de Navarra, San Sebastián 20018, Spain; b Instituto de Ciencia de Datos e Inteligencia Artificial (DATAI), Universidad de Navarra, Pamplona 31080, Spain

**Keywords:** MoA, Deep learning, Explainable artificial intelligence, Drug response prediction

## Abstract

**Background:**

Although Deep Neural Networks (DDNs) have been successful in predicting the efficacy of cancer drugs, the lack of explainability in their decision-making process is a significant challenge. Previous research proposed mimicking the Gene Ontology structure to allow for interpretation of each neuron in the network. However, these previous approaches require huge amount of GPU resources and hinder its extension to genome-wide models.

**Methods:**

We developed SparseGO, a sparse and interpretable neural network, for predicting drug response in cancer cell lines and their Mechanism of Action (MoA). To ensure model generalization, we trained it on multiple datasets and evaluated its performance using three cross-validation schemes. Its efficiency allows it to be used with gene expression. In addition, SparseGO integrates an eXplainable Artificial Intelligence (XAI) technique, DeepLIFT, with Support Vector Machines to computationally discover the MoA of drugs.

**Findings:**

SparseGO's sparse implementation significantly reduced GPU memory usage and training speed compared to other methods, allowing it to process gene expression instead of mutations as input data. SparseGO using expression improved the accuracy and enabled its use on drug repositioning. Furthermore, gene expression allows the prediction of MoA using 265 drugs to train it. It was validated on understudied drugs such as parbendazole and PD153035.

**Interpretation:**

SparseGO is an effective XAI method for predicting, but more importantly, understanding drug response.

**Funding:**

The Accelerator Award Programme funded by 10.13039/501100000289Cancer Research UK [C355/A26819], Fundación Científica de la AECC and Fondazione AIRC, Project PIBA_2020_1_0055 funded by the 10.13039/501100003086Basque Government and the Synlethal Project (RETOS Investigacion, Spanish Government).

**Research in context**

**Evidence before this study**

Drug response requires a thorough understanding of its mechanism of action (MoA). Current studies of MoA involve high-dimensional profiling techniques, and there are limited approaches that use computational prediction. Deep neural networks have demonstrated their potential in predicting drug response. Some of them (DrugCell) use the Gene Ontology hierarchy to understand the roles of the inner nodes of the neural network. Using this information, the MoA of some drugs is predicted and is concordant with previous knowledge. Unfortunately, these networks require large amounts of GPU memory that limits its use in genome-wide -omics.

**Added value of this study**

The first added value of this study is methodological: SparseGO makes very efficient use of GPU resources (both in terms of memory and computing power). It drastically reduces the time to train the network and, more importantly, permits the use of full-genome information without exhausting the GPU memory. With the ability to use a very large number of omics variables as input, SparseGO demonstrates remarkable flexibility in mimicking any ontology, enabling it to accurately predict drug response and MoA. In addition, the use of an explainable artificial intelligence technique, DeepLIFT, improves the discovery of the MoA and provides insights into the contributions of individual features and neurons to the model's output. Its performance regarding the prediction of drug efficacy is demonstrated through testing on diverse datasets and using different cross-validation techniques. Its performance in the prediction of MoA is also systematically computed using a cross-validation scheme. The accessibility and promising performance make it a valuable tool for researchers and clinicians alike.

**Implications of all the available evidence**

SparseGO can predict drug response and MoA, helping to accelerate the development of effective treatments and improve patient outcomes. The structure can be modified to suit researchers' needs and, using expression data, our model can even be used for drug repositioning. The results also show that it is still difficult to model the efficacy of a drug using only their chemical characteristics. As we continue refining and improving it, we believe it has the potential to replace traditional drug sensitivity tests or even lead to the development of drugs.

## Introduction

The rise of -omics and clinical data, coupled with the urgency to find unambiguous solutions for patients, has boosted the use of deep learning algorithms in the biomedical world. The problem now is not finding “the cure” for cancer, but the optimal treatment for a patient given their genome and clinical history. Deep learning (DL) algorithms collect these huge amounts of clinical data and provide very accurate solutions by establishing relationships between the neurons in a network. However, in a clinical context, an excellent prediction is not enough. Understanding a drug’s mechanism of action (MoA) is essential for several reasons, such as determining the adequacy of dose and latency of response, identifying which patients are most likely to respond to it, and understanding its side effects.^1^, ^2^, ^3^ Knowing the MoA can even lead to the development of drugs and strategies for combination therapies.^1^, ^2^, ^3^

Current MoA studies involve high-dimensional profiling techniques.^2^^,^^4^ These methods generally involve the preparation of treated and untreated sample sets, profiling or screening of the samples, filtering of pertinent information, and comparison of information between states, making them time-consuming and requiring high-throughput technologies.^2^^,^^5^ Although these techniques are now being combined with AI methods to interpret the results, researchers still need to devote a lot of resources to developing the right assay conditions, avoiding technical errors, and identifying key readouts.^2^ Efforts should be made to develop methods based mainly on computation to provide the MoA of a drug with fewer resources.

With this aim, the Ideker Lab introduced the concept of visible neural networks^6^ (VNNs). VNNs are algorithms that structure deep neural networks using knowledge of human cell biology. They later built DrugCell,^7^ a VNN structured according to the Gene Ontology (GO) hierarchy that simulates the response of human cancer cells to drugs and reveals the most important biological processes involved in the response to a particular drug. The model can estimate with good precision the efficacy of a compound using as input the mutational status of a cell line (using 3008 genes) and the Morgan fingerprint^8^ of the drug. In the model, each subsystem (GO term) of the hierarchy is represented by a layer of the VNN that includes its child subsystems and its directly annotated genes.^7^ The model can accurately predict drug response. However, the implementation limits its computational efficiency. Improving the economy of computational resources would allow larger numbers of features and samples which, in turn, result in more descriptive models.

Additionally, DrugCell’s customized method for interpreting the network -Relative Local Improvement in Predictive Power (RLIPP)- has been validated only in some drugs. The model used the RLIPP score to predict the MoA of drugs with the computed weights of the VNN. The RLIPP compares the performance of two L2-penalized linear regression models for predicting the drug response: one uses the weights of a GO term as parameters and the other uses the weights of its children. The method indicates if the weights of a term are more important than those of their children.^6^ If this is the case, the origin of the improvement is the nonlinearity of the transfer function of a neuron in the network. RLIPP only considers the node weights of the parent and its children, and not the relationships between all nodes. Furthermore, although the method is well described, its readily implementation is not publicly available.

Intending to obtain a more efficient and descriptive model, Huang et al. developed ParsVNN.^9^ This method prunes redundant components of the DrugCell model and builds highly parsimonious and interpretable sparse models. The pruning is guided by cell line specific training data, resulting in cancer-specific models that require low storage memory and low prediction time. The models, however, can only be used to study one type of cancer, and the pruning process is computationally expensive. In addition, as it occurs for DrugCell, the incorporation of more genomic data (such as more mutations, gene or even isoform expressions) is not straightforward since it is limited by current specifications of hardware.

The goal of this work is to build a competent and biologically interpretable neural network that 1) provides accurate drug response predictions for any cancer cell line, 2) uses any chosen set of mutations or expression data, 3) optimizes GPU memory requirements, 4) optimizes training and testing time, and 5) provides accurate explanations of the network decisions.

To accomplish the last objective, we explored eXplainable Artificial Intelligence (XAI), a branch of machine learning (ML) that attempts to explain why algorithms make their decisions.^10^ Attribution methods^11^, ^12^, ^13^ are algorithms that rank the importance of the nodes in a network to get a specific result and thus open the “black box.” In addition, since networks are interpretable, their behaviour can be validated by humans which, in turn, increases confidence in the results.^14^ In this case, to predict the MoA of drugs, we propose to use the DeepLIFT^11^ attribution method, an XAI approach to identify which neurons in the network are critical for making a prediction.

SparseGO meets all these objectives. Using sparse layers, it reduces the amount of computational resources needed. As a result, the input layer can be much larger: we have tested it using both mutations (3008 genes) and expression (around 15,000 genes). The results using mutations demonstrate remarkable similarity (with a favourable advantage for SparseGO) to those of a state-of-the-art model (DrugCell) when using an identical input layer and require only a fraction of the time to train the network. To improve generalization and accuracy, we trained another model with an additional dataset and tested it on a separate dataset. Additionally, the use of expression data increases the performance of the model and enables it to be used for drug repositioning. We also developed a method named DeepMoA that accurately predicts a drug's MoA using DeepLIFT findings and MoA annotations collected from the ‘ChEMBL protein target slim’^15^ and the Cancer Therapeutics Response Portal v2 (CTRPv2).^16^ The accuracy of the MoA prediction method was computationally tested on 265 drugs (using a train-validation-test scheme). To confirm that our algorithm can infer how drugs exert their therapeutic effects, we also examined the results for some non-annotated drugs and found that they agree with those obtained from in vivo tests or other studies in the literature.

## Methods

### SparseGO’s architecture

**Fig. 1:**
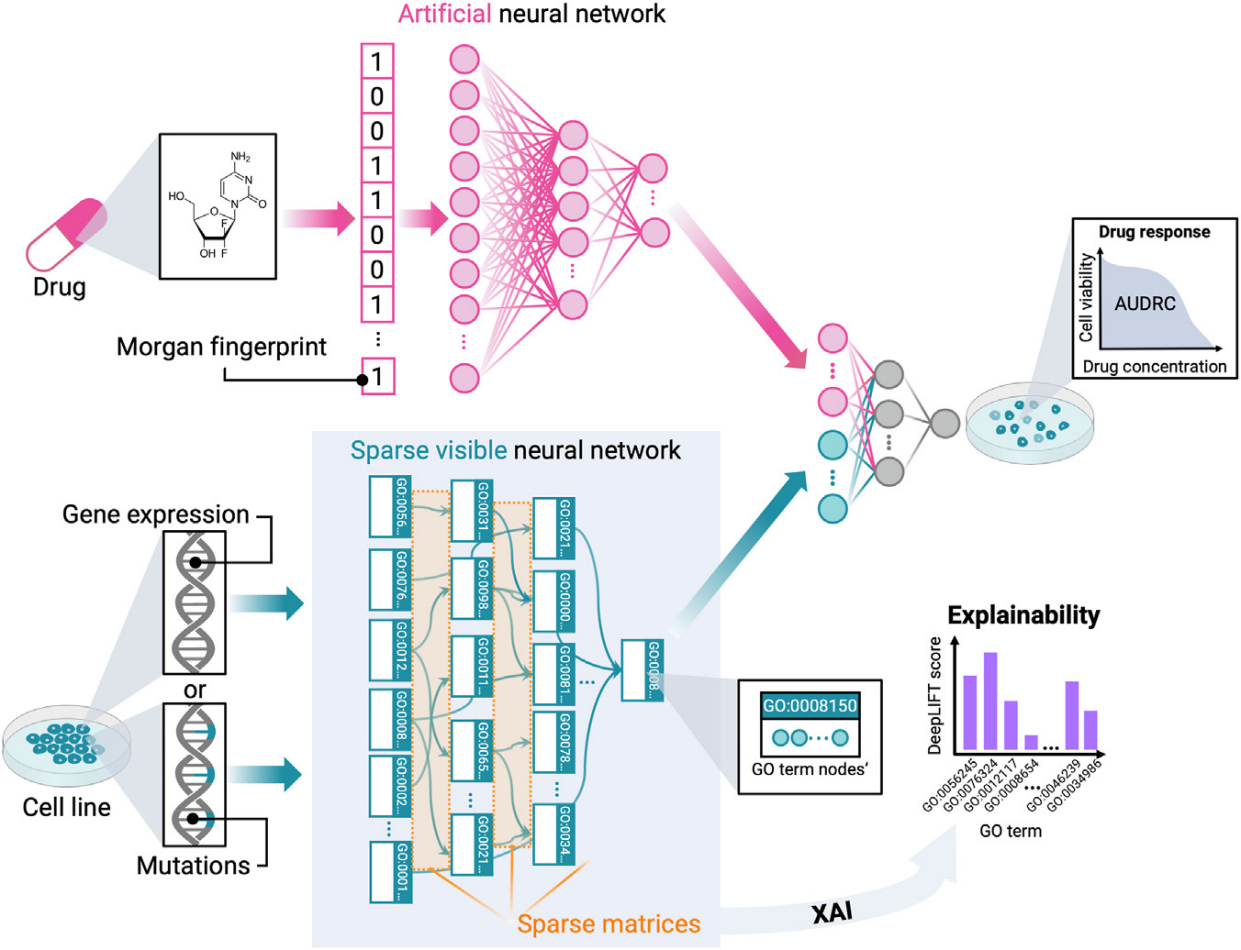
SparseGO architecture. The network has two branches: an artificial neural network that takes the Morgan fingerprint of a drug as input, and a sparse VNN that takes the gene expression or mutations of a cell line as input. In the VNN, each GO term is represented by “k” nodes, where the hyperparameter “k” is set to 6 (in all cases). The connections between the layers of the hierarchy are represented using sparse matrices. The output of the network corresponds to the cell line’s viability, measured by the area under the dose-response curve. To assess the significance of the VNN nodes, we employed the DeepLIFT method.

SparseGO was designed based on DrugCell’s two-branch structure, which includes a VNN that captures the hierarchical relationships of the GO graph, and an Artificial Neural Network (ANN) that integrates the Morgan fingerprint^8^ of the compounds (Fig. 1). It is important to note that while the ANN architecture in SparseGO is comparable to DrugCell, the layers of our VNN differ significantly.

To generate a response for a specific drug, the output of both branches is combined and integrated into another fully connected network. The predicted continuous value represents the area under the dose-response curve (AUDRC) normalized such that AUDRC = 0 represents complete cell death, AUDRC = 1 represents no effect, and AUDRC > 1 represents that the treatment favours cell growth.^7^

SparseGO uses a sparse matrix representation to depict the connections of the GO hierarchy. A matrix is sparse if most of its entries are null. There are different methods to store sparse matrices. All of them (if the proportion of null entries is large) require less memory to store and are more efficient when performing computations (since zeros can be skipped in many operations). This matrix representation was used to create a neural network of sparse linear layers.

Using this architecture, we developed three models. The first model closely resembles DrugCell, utilizing mutations as the input. The other two models incorporate either mutations or gene expression as inputs, with some modifications implemented to enhance the prediction of drug response.

### The VNN branch

The structure of the VNN shown in Fig. 1, is created by known parent-child relationships drawn from a domain of the GO hierarchy. The lowest level of the hierarchy contains the genes, and the higher levels contain the GO terms, starting from the most specialized terms to the most general (or root term). Genes are linked only to the terms in which they are annotated. To maintain a reasonable size of the hierarchy, we imposed that each term be annotated to a minimum number of genes (to avoid overly specific terms) and that a minimum number of those genes be different from those of their children (to avoid parents with genes annotated nearly identical to those of their children). Additionally, the network can only have a limited number of parent-child relations above the bottom layer subsystems. These three parameters are user-defined. If a term does not meet the criteria, it is deleted, and its children terms and annotated genes are assigned to their parent terms.

To ensure a fair comparison, we adopted the ontology used in DrugCell for the two mutation models, which was created similarly. For the expression model, terms were retained if they had at least 5 annotated genes and at least 10 genes different from those of their children (if they have any). Finally, the hierarchy was restricted to a maximal depth of 8 subsystems. Furthermore, as depicted in Fig. 1, each GO term is represented by a set of k neurons, enabling it to encompass a diverse range of values. In all models, we employed six neurons (chosen using hyperparameter tuning) to define each GO term.

**Fig. 2:**
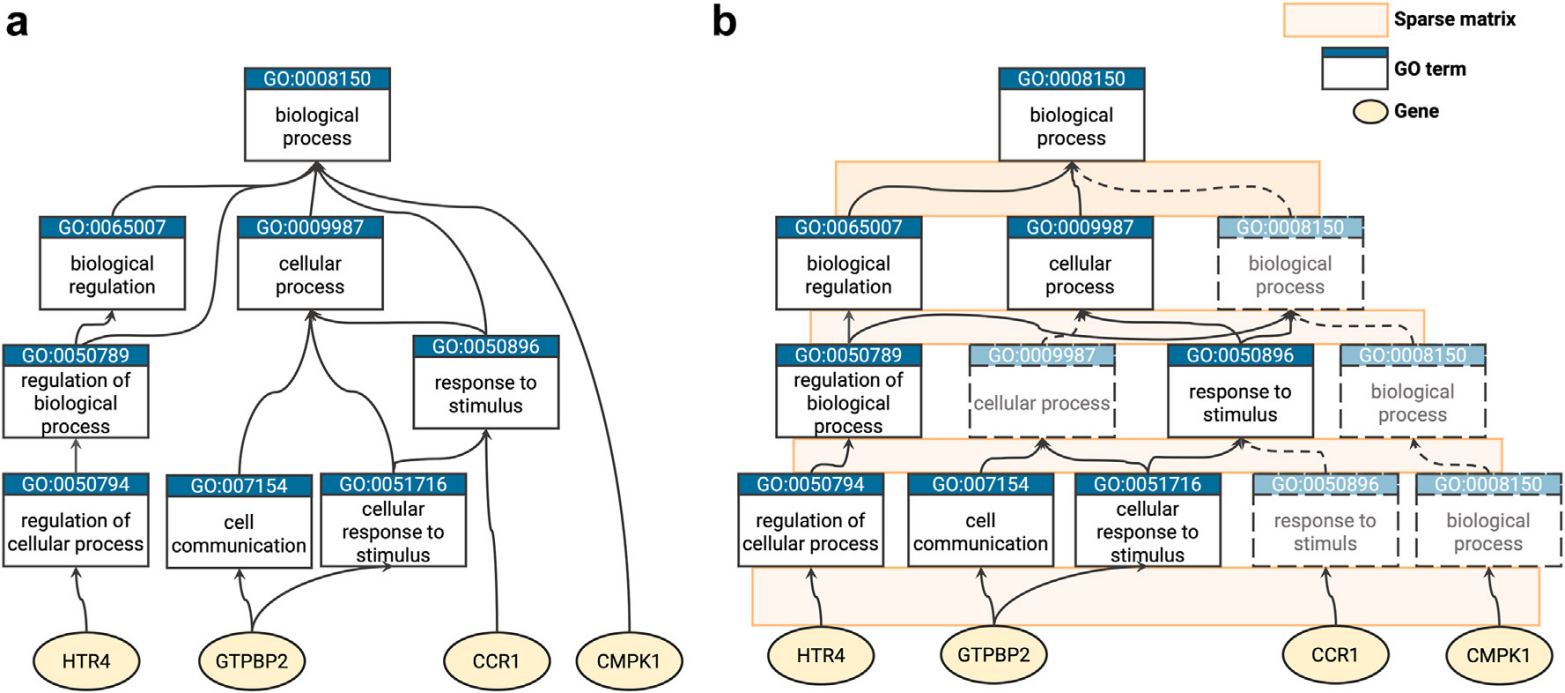
Example of hierarchy. (a) Example of part of a hierarchy structured as in GO. (b) Hierarchy “a” with the addition of “virtual nodes”, dashed lines represent virtual connections and dashed terms represent “virtual nodes”. In the network, connections between layers are represented with sparse matrices. No connection are allowed between non-adjacent layers.

Given that the GO is structured as a directed acyclic graph, it is important to consider that a GO term can have relationships with parent terms that are not directly above it. SparseGO uses sparse matrices that represent layer-layer connections. To overcome the constraint of terms being connected only to adjacent layers, we introduced virtual nodes as needed to establish connections between terms that are not directly adjacent. Fig. 2a is a subset of the GO hierarchy and Fig. 2b shows the same network including some virtual terms to represent the same ontology using only connections between adjacent layers.

The output of each layer of the VNN is represented by the vector *O*^*(s)*^:

$$O^{\left(s\right)}=f\;\left(W^{\left(s\right)}I^{\left(s\right)}+b^{\left(s\right)}\right)$$

For the bottom connected levels (first layer), *W*^(s)^ represents the sparse weight matrix of dimensions (*k∗p) x g*, *k* is the number of neurons assigned to each subsystem, *p* is the number of terms on the parent level, and *g* the total number of genes. The weight vector *b*^*(s)*^ has dimension (*k∗p).* For this layer, the input vector *I*^*(s)*^ is the mutational state or gene expression vector. The function *f* is a non-linear transformation based on the hyperbolic tangent.

For all the other layers, *W*^(s)^ represents the sparse weight matrix of dimensions (*k∗p) x* (*k∗c)* and *b*^*(s)*^ the weight vector of dimension (*k∗p)*, where *p* is also the number of terms on the parent level and *c* the number of terms on the child level. The input vector *I*^*(s)*^ represents the output of the previous layer. There are additional batch normalization layers that standardize the inputs making the training process faster and more stable.

**Fig. 3:**
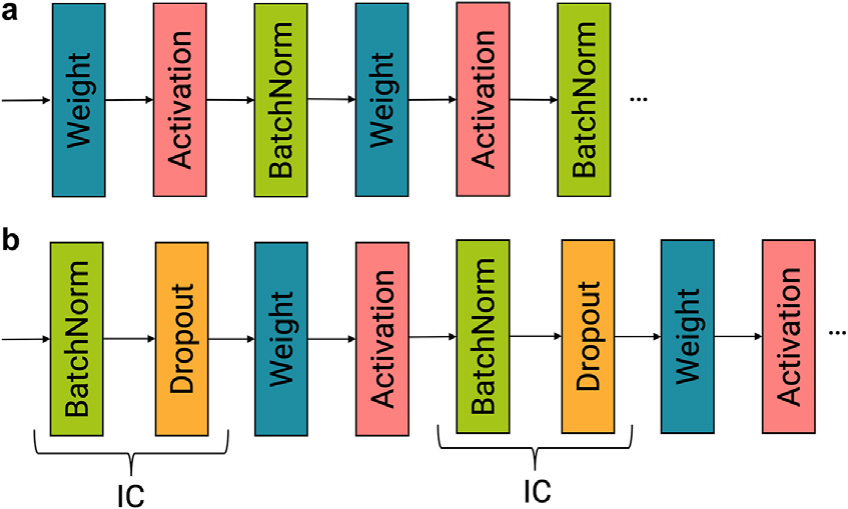
(a) Layer order similar to the DrugCell model, the batch normalization layer is placed after the activation layer. (b) Layer order as suggested by Chen et al. the IC layer is placed before the weight layer.

In one of the mutation models, the batch normalization layers are positioned after the activation layers (Fig. 3a), to resemble DrugCell. However, experimental results indicated that incorporating the Independent-component (IC) layer developed by Chen et al.^17^ led to improved performance by reducing overfitting. This IC layer combines Batch Normalization and Dropout and is placed before the weights layer (Fig. 3b). The purpose of this layer is to enhance the stability of the training process, accelerate convergence speed, and improve generalization performance.^17^

### The drug’s branch

The ANN architecture of the mutation models consists of three fully connected hidden layers, comprising 100, 50, and 6 neurons, respectively. In contrast, as the gene expression models have a larger input in the VNN, the size of the VNN output branch is set to 30 instead of the 6 neurons used in the mutation models. Consequently, the size of the ANN is increased to 200, 100, and 50 neurons. As illustrated in Fig. 1, the last neurons of the final layer in the ANN are concatenated with the last neurons of the final layer in the VNN. These concatenated neurons are then connected to an additional layer of neurons, which ultimately feed into a final layer consisting of a single neuron. The activation function used in these layers is the hyperbolic tangent, and depending on the approach, batch normalization layers or IC layers are also included.

Table 1 summarizes the dimensional characteristics of the layers in the models.

**Table 1:** Characteristics of the model layers depending on the input data.

	VNN input size	GO terms	Neurons per GO term	Drug branch layer sizes	Output neurons (final VNN layer)	Output neurons after concatenation (before the output)
Mutation models	3008	2048	6	100, 50, 6	6	6
Expression model	14,457	4840	6	200, 100, 50	30	40

VNN input size specifies the total number of features that represent the cell lines. GO terms represents the number of GO terms used to create the ontology. Neurons per GO term represents the number of neurons associated with each GO term. Drug branch layer size denotes the sizes of the layers in the drug branch. Output neurons (final VNN layer) indicates the number of output neurons in the final VNN layer, and Output neurons after concatenation (before the output) specifies the number of output neurons in the layer obtained by concatenating both the drug and VNN branches of the model.

### Data acquisition and processing

The model presented relies on two input vectors: the mutations or expression data of the cell line and the Morgan fingerprint of the drug. With these inputs, the model predicts the drug response, which is measured as the AUDRC. The AUDRC values are calculated from raw data obtained through screening experiments. The process of data acquisition is described below.

#### Drug sensitivity datasets

Traditionally, many machine learning algorithms employed for personalized cancer treatment rely on CV within a single study to assess the accuracy of their models. However, as studied in,^18^ it has been well-documented that there are inconsistencies in genomic and response profiling across different studies. Consequently, relying solely on training with a single study may lead to an overestimation of the prediction performance.^18^

To address this issue, the DrugCell model was trained using 509,294 cell line-drug pairs from two publicly available datasets: CTRPv2 and Genomics of Drug Sensitivity in Cancer 1 (GDSC1). To make a fair comparison, we trained one of the mutation models using the same data. However, in the rest of the study, we took a different approach. We developed joint models by incorporating three publicly available datasets: CTRPv2, GDSC1, and Genomics of Drug Sensitivity in Cancer 2 (GDSC2). In addition to conducting CV tests, we evaluated the generalization ability of our models by predicting on an independent dataset known as Profiling Relative Inhibition Simultaneously in Mixtures (PRISM).^19^ This dataset contains non-oncological drugs tested on cancer cell lines. This evaluation enabled us to assess the performance of our models on an entirely disparate dataset, thereby gauging their effectiveness in handling unseen data.

The characteristics of the drug response datasets used is shown in Table 2. We included only the cells and drugs for which we were able to obtain their corresponding genomic data from the Cancer Cell Line Encyclopedia (CCLE)^20^ and their Simplified Molecular Input Line Entry System (SMILES) representations, respectively. We also excluded dose response samples that had missing concentrations or viability values for a given dose.

**Table 2:** Characteristics of drug response studies used.

Study	Cells with available CCLE genomic data	Drugs with available SMILES	Dose response pairs	Viability assay
CTRPv2	793	544	351,922	CellTitreGlo
GDSC1	620	294	191,585	Resazurin or Syto60
GDSC2	539	172	135,993	CellTitreGlo
PRISM	453	1437	690,995	PRISM Repurposing assay

#### Morgan fingerprint encoding

To represent the chemical structure of drugs, we obtained the SMILES notation for each compound from PharmacoGX^21^ and PubChem,^22^ if no initial matches were found we manually annotated them. Then, we calculated the Morgan fingerprint (radius = 2) using RDKit (http://www.rdkit.org/), which was then hashed into a bit vector of length 2048 for model training. The same methodology was employed for training DrugCell.^7^

#### Genomic data processing

For the mutation models, we used the same genomic data as DrugCell. They obtained non-synonymous coding mutations previously annotated by the CCLE and filtered the dataset to represent only the top 15% most frequently mutated genes (n = 3008). They represented each cell-line genotype as a bit vector indicating the mutational status of each gene in that cell line (0 = wild type; 1 = mutated).^7^

To obtain the gene expression data, we used the Transcript Per Million (TPM) 22Q2 RNAseq gene expression dataset (TPM_22Q2) also created by the CCLE. This dataset includes data from 19,221 genes, 1406 cell lines, 33 primary diseases and 30 lineages. Expression is represented as the numerical protein coding gene expression change at scale (log2 (TPM + 1)). We only used the expression values of genes that had Gene Ontology annotations so that we could incorporate them into the sparse VNN. In total 14,457 genes were used for each cell-line.

#### Drug response integration

During drug screening experiments, raw data is collected to measure the survival or viability of cells at different drug concentrations. This serves as the basis for calculating various metrics such as IC_50_, AUDRC, or AADRC (area above the drug response curve), which provide insights into the drug's efficacy and potential toxicity.

In the mutation model created for comparison with DrugCell, we employed the same AUDRC calculation method as DrugCell, referred to as AUDRC1. This method involved applying the trapezoidal rule to integrate the dose-response curve. The minimum and maximum concentrations considered for each experiment were determined based on the specific experiment's range.

However, we encountered a limitation when comparing data from different experiments that tested the same drug at varying doses. The former approach led to counterintuitive results, where experiments with identical outcomes for overlapping concentrations yielded different AUDRC1 values simply due to differences in their maximum and minimum concentration ranges.

To address this limitation and facilitate comparison across the different datasets used, we slightly modified it. We standardized the minimum and maximum concentrations for all drugs to 100 pM and 100 μM, respectively. Instead of relying on the trapezoidal rule, we employed a four-parameter logistic regression model using the dr4pl package.^23^ This allowed us to accurately model the dose-response curve and numerically integrate the fitted curve to calculate the AUDRC. This alternative approach (AUDRC2) demonstrated a slight improvement in the correlation between datasets compared to the trapezoidal rule (these results are not presented here).

Table 3 presents the key characteristics of the datasets used for training and testing the models.

**Table 3:** Overview of the characteristics of the datasets created for training and testing the different models.

Dataset	Studies included	Total dose response pairs	Drug response metric	Drugs	Cell lines	Features
DrugCell	CTRPv2, GDSC1	509,294	AUDRC1	684	1125	Mutations
SparseGO (mutations)	CTRPv2, GDSC1, GDSC2	695,501	AUDRC2	790	885	Mutations
SparseGO independent test (mutations)	PRISM	688,446	AUDRC2	1432	459	Mutations
SparseGO (expression)	CTRPv2, GDSC1, GDSC2	679,500	AUDRC2	790	852	Expression
SparseGO independent test (expression)	PRISM	690,995	AUDRC2	1432	461	Expression

Studies included lists the studies that were incorporated. Total dose response pairs indicates the total number of sets of dose-response samples obtained from the combined studies, including duplicate pairs if present. Drug response metric specifies the metric used to measure the drug response. Drugs represents the number of unique drugs included in the dataset. Cell lines indicates the number of unique cell lines included in the dataset. Features describes the type of features associated with the cell lines in that particular dataset.

### Models training and evaluation

#### Cross-validation schemes

To ensure model generalization and robustness, we employed a five-fold cross-validation approach. Four groups were used for training, while the remaining group was reserved for testing. Additionally, from the training data, a subset of samples was extracted for validation purposes. We adopted three different methods of separating the data for the CV folds. Two of these approaches focus on evaluating the model’s ability to predict drug sensitivity data for previously unseen cell lines and compounds. These tests are particularly challenging as they assess the model's performance on completely different data.

The dose response pairs from CTRPv2, GDSC1, and GDSC2 were merged to create a unified dataset, which was used for the CV. For further testing, we evaluated the generalization performance of our models by using the PRISM dataset.

**Fig. 4:**
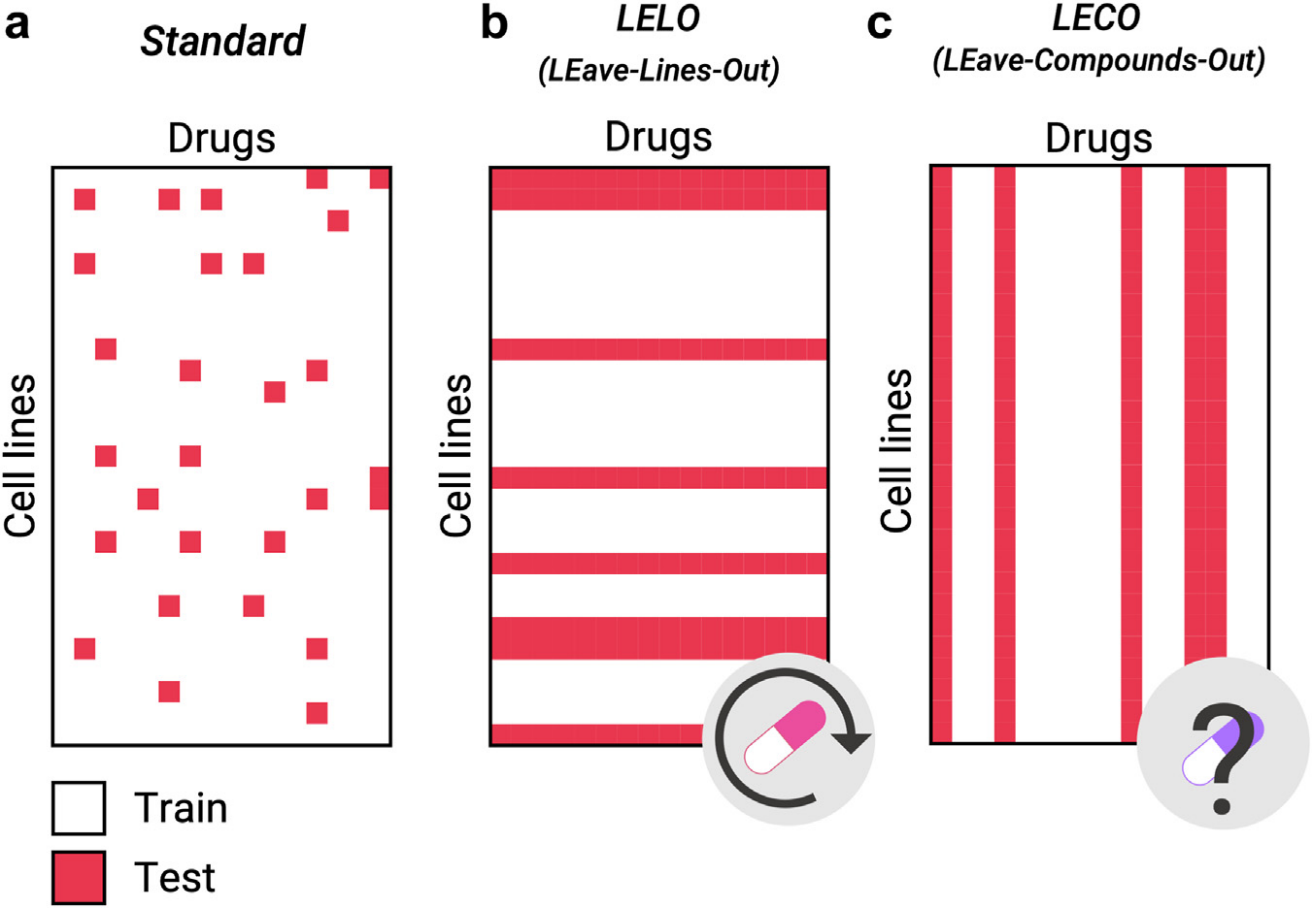
Cross-validation approaches. Examples of the different approaches to estimating model performance. (a) In the standard approach, cell line-drug pairs are randomly selected. (b) In the LELO approach, some cell lines are chosen for training and the rest for testing. This method simulates a drug repositioning experiment. (c) In the LECO approach, some compounds are chosen for training and the rest for testing. This approach provides unbiased estimates of the predicted AUC when predicting the effect of an unseen drug on a cell line.

1) *Standard approach:* The standard approach involves randomly splitting the dose response pairs in K groups. One is removed from the training set and used for prediction (Fig. 4a). This approach predicts the performance of drugs (within the training set) when used on cells (also within the training set), even if the specific combination of drug-cell line is not included in the training set. This approach was employed by DrugCell.

To perform a similar test using the independent dataset (PRISM), we selected drugs and cell lines that were common to both the training dataset and PRISM. We then predicted the AUDRC2 for these drugs in PRISM using our fully trained model (trained with all the available samples).

2) *LEave-Lines-Out (LELO):* Here, cell lines are split into K groups. In this case, the model is trained with some cell lines and predicts the effect of drugs in cell lines excluded from the training set (Fig. 4b). This scenario is designed to mimic situations where the model is employed to extrapolate the data to unseen cell lines, making it particularly suitable for drug repositioning purposes.

As we could not find data from different cell lines for PRISM, we did not perform a test with the independent dataset.

3) *LEave-Compounds-Out (LECO):* In this approach, drugs are split into K groups. The model is trained with some drugs and predicts the drug response in the cell lines for drugs not used in the training set (Fig. 4c). This approach is ideal for in-silico drug sensitivity testing. However, as compounds have very different response patterns and chemical characteristics, this scenario is more challenging compared to the previous ones.^18^

In the case of the independent dataset test, we selected drugs from the PRISM dataset that were not included in our training dataset (1144 drugs). Then, we used our fully trained model to predict the AUDRC2 values for these selected drugs.

#### Parameters of the model

The cost function used for SparseGO training is the mean squared error between the measured and predicted AUDRC, with the standard gradient descent optimization procedure using a momentum of 0.9. The models were trained with a decay rate of 2e-3 and a learning rate of 0.1. In the models that included dropout layers, a dropout rate of 15% was applied, which randomly deactivated 15% of the neurons. The performance of the models was measured using Pearson’s correlation between the actual and predicted drug responses in the test data. All training runs used 400 epochs and a batch size of 20,000 samples. We performed hyperparameter tunning to choose the best model parameters. Training was performed with a maximum of 15 GB of RAM and an NVIDIA GeForce RTX 3090.

### Identification of drug-specific mechanism of action

The primary aim of SparseGO is to ensure interpretability by understanding the MoA of drugs. To achieve this, we devised a methodology that uses SVMs models generated from the output of the XAI algorithm, DeepLIFT. For training the models, we leveraged MoA labels for some compounds extracted from the ‘ChEMBL protein target slim’ and CTRPv2 datasets.

DeepLIFT (Deep Learning Important FeaTures) is a method that compares the activation of each neuron with a reference activation and assigns contribution scores according to the difference.^11^ In this case, the reference activation chosen was the median of the expression data and the fingerprint of glucose. We chose glucose because we wanted to contrast the drug’s effect with that of a cellular-beneficial substance. DeepLIFT backpropagates “the contributions of all neurons in the network to every feature of the input”.^11^ Both the input layer and the internal layers are given an attribution score. DeepLIFT addresses the saturation problems that appear on other perturbation-based and gradient-based approaches.^6^ We used DeepLIFT to compute scores for all neurons of SparseGO’s VNN branch by introducing the gene expression of all cell lines and the drugs to the trained SparseGO model.

First, to assess whether the DeepLIFT scores were informative for MoA identification, we followed the visualization approach used by Jang et al.^5^ that mapped drug scores in a low-dimensional vector space. To visualize the DeepLIFT, we used the T-SNE algorithm.^24^ This technique allowed us to reduce the dimensionality of the DeepLIFT scores and represent them in a visually interpretable format. We retrieved known classifications for certain drugs, such as inhibitor types, from clue.io (https://clue.io/). Then, we selected and plotted those drugs whose classification was shared by 2 or more drugs. To identify clusters within the vector space and gain insights into the relationships between drugs, we employed mclust,^25^ an R package that facilitates model-based clustering using finite Gaussian mixture models. By applying this clustering algorithm, we were able to detect meaningful patterns and groupings in the visualized DeepLIFT score data.

**Fig. 5:**
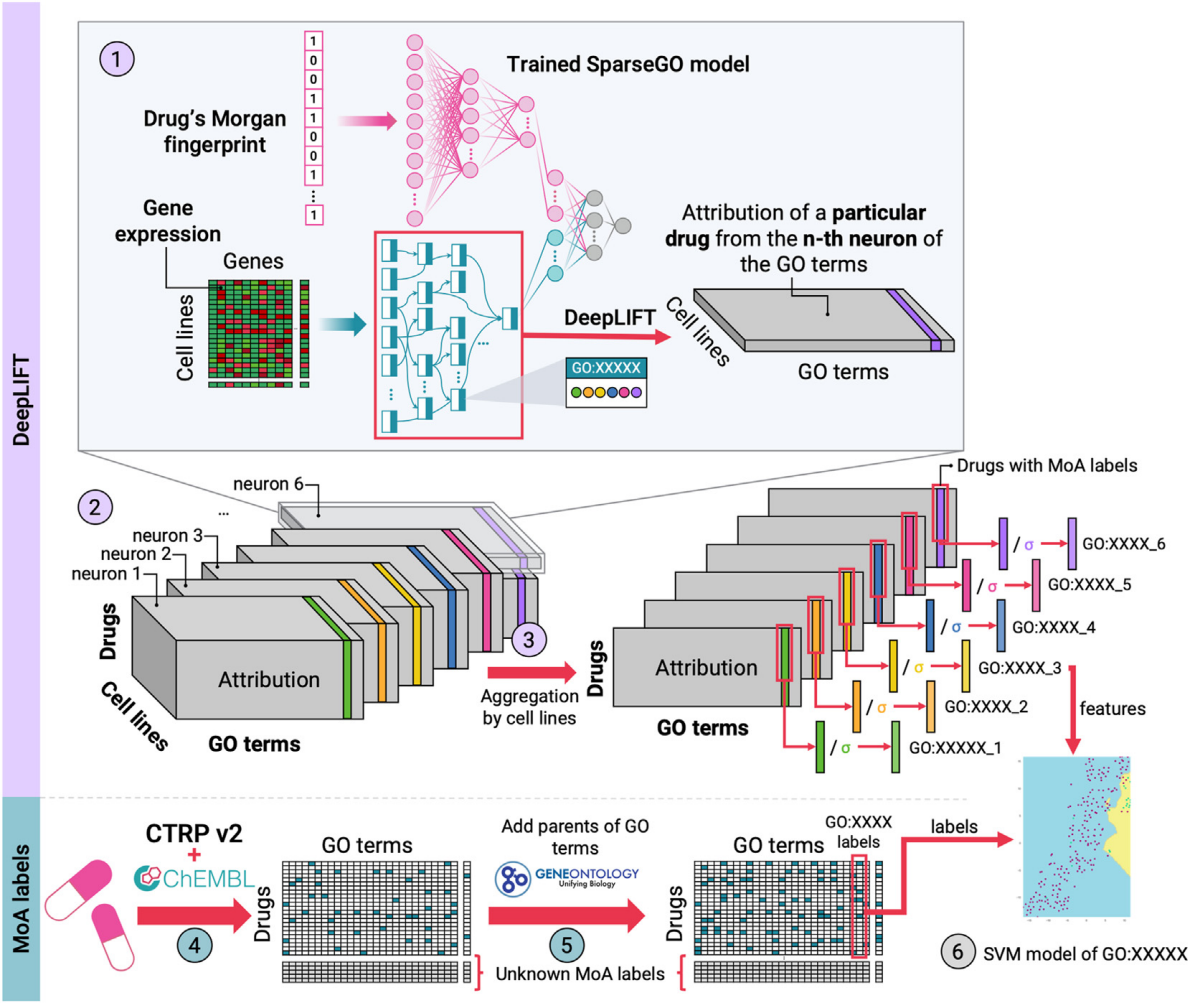
DeepMoA framework with a toy example of how a model is created. 1) The trained SparseGO model is fed a drug’s Morgan fingerprint and gene expression data from all cell lines. DeepLIFT determines the significance of the n-th neuron of each GO term for each cell line for a particular drug (the one corresponding to its Morgan fingerprint). This process is repeated for each drug changing the Morgan fingerprint accordingly. The purple array corresponds to the attribution of the 6th GO:XXXXX neuron of all cell lines for a given drug. 2) The resulting 3D tensors include the GO term attribution for each cell line-drug pair. Each GO term is represented by six neurons, resulting in six 3D tensors. The colored matrices represent the attribution of GO:XXXXX. 3) The scores for each drug are aggregated by adding the scores of all cell lines. Then, the attribution of each of the neurons of a term is divided by its standard deviation. The colored arrays after normalization are the features that will be used to predict whether GO:XXXXX is one of the MoAs of a drug. 4) The MoA labels are extracted from ChEMBL and CTRPv2. Some drugs are not annotated (unknown MoA labels). 5) MoA labels are extended upward, i.e., if a drug is annotated to a GO, it will also be annotated to all its ancestors. 6) Finally, for each GO term, an SVM model is created using the features and labels for drugs with known MoA. The two-dimensional representation of the SVM decision boundary of GO:XXXXX is shown.

After ensuring that the DeepLIFT scores could provide relevant insights, we developed the DeepMoA method. The framework of the method is depicted in Fig. 5. First, the trained SparseGO model is fed with the fingerprint of a drug as well as the gene expression of all cell lines (section 1 of Fig. 5). Next, the VNN branch is subjected to the DeepLIFT algorithm, which calculates the significance of the GO terms for each cell line. This procedure is repeated for each of the six neurons representing each GO term. In the end, each cell line has six importance scores per each GO term. The process is performed for all drugs by varying the Morgan footprint of the input vector. The resulting 3D tensors include the GO term attribution for each cell line-drug pair (section 2 of Fig. 5). As noted, each GO term is represented by six neurons, resulting in six 3D tensors.

We then added the cell line scores to maintain a global view of the importance of neurons, which results in six attribution scores per GO term for each drug (section 3 of Fig. 5). Then, the attribution of each of the neurons of a term is divided by its standard deviation. These scores were used as input parameters (independent variables) of the SVM models.

On the other hand, we obtained the MoAs to be predicted (dependent variables) of the models from ‘ChEMBL protein target slim’ and CTRPv2. ‘ChEMBL protein target slim’ is a GO-based tool that collects biological information about drug target proteins from ChEMBL.^26^ First, from all the drugs, we selected those that were annotated in ChEMBL and extracted their target proteins (if known). The tool provides the GO terms annotated to those proteins.^15^ Not all target proteins have GO terms associated with them. In contrast, the CTRPv2 database already contains GO annotations for some drugs, so we simply extracted them. Then, we extended all the annotations upward, i.e., if a drug is annotated to a GO, it will also be annotated to all its ancestors. As a result, we obtained a matrix containing the annotated GO terms (MoA labels) of some drugs.

Finally, we tested whether DeepLIFT attribution scores could predict the MoA labels using RBF Kernel SVM models. Fig. 5 describes a simplified example of how the SVM decision boundary is built for a GO term, in which the scores of the six GO:XXXXX neurons are used as parameters to determine whether a drug has GO:XXXXX as its MoA. We set the RBF kernel parameters C and γ to 1 and the inverse of the number of features times the variance of the features (suggested values by scikit-learn^27^), respectively. Using different values did not improve the performance. To address class imbalance, we added weights to the regression inversely proportional to the class frequencies (again, as suggested by scikit-learn). We scaled the output of SVM models to probability values via Platt scaling. When using Platt scaling, SVM outputs are transformed into probabilities by tuning the parameters of a sigmoid function.^28^ The adoption of Platt scaling was essential as we required probabilities to compare the performance across different drugs.

Using this information, we built SVM models for all GO terms that were annotated in at least 16 drugs (fewer annotations do not allow the creation of accurate models). To properly evaluate the models' performance, we used a 4-fold cross-validation method. We divided the drugs into 4 groups, 3 for training and 1 for testing at each fold. Then we computed the AUROC for each SVM model to compare the true MoA labels of a GO term with the predicted probabilities of the same GO term (performance by GO terms) and the AUROC to compare the true MoA labels with the predicted probabilities of each drug (performance by drugs).

### Feature importance

The DeepLIFT method assigns an attribution score to all neurons including the input data. In our study, it is challenging to interpret how the individual elements of the Morgan fingerprint relate to specific substructures of the molecule, thereby limiting the informativeness of the scores derived from it. In contrast, for the cell lines, each feature directly corresponds to a gene, which makes it easier to comprehend their significance. By applying DeepLIFT, we extracted the scores associated with each gene and analysed their importance in predicting the AUDRC2.

As before, the trained SparseGO model takes as input the drug's fingerprint and the gene expression of all cell lines. By adding the cell line scores, we obtained a score for each gene associated with each drug. This analysis allowed us to investigate the significance of individual genes in predicting the AUDRC2 for a specific drug.

### Role of funders

Funding sources were not involved in the development of this work.

## Results

In this section, we present the findings of our study using SparseGO. First, we demonstrate the superior computational performance of SparseGO. Additionally, we compare the results of SparseGO using mutations and gene expression as input under the three CV schemes. Lastly, we showcase the interpretability of the model achieved through the application of XAI techniques.

### SparseGO outperforms DrugCell in computational resource efficiency

**Fig. 6:**
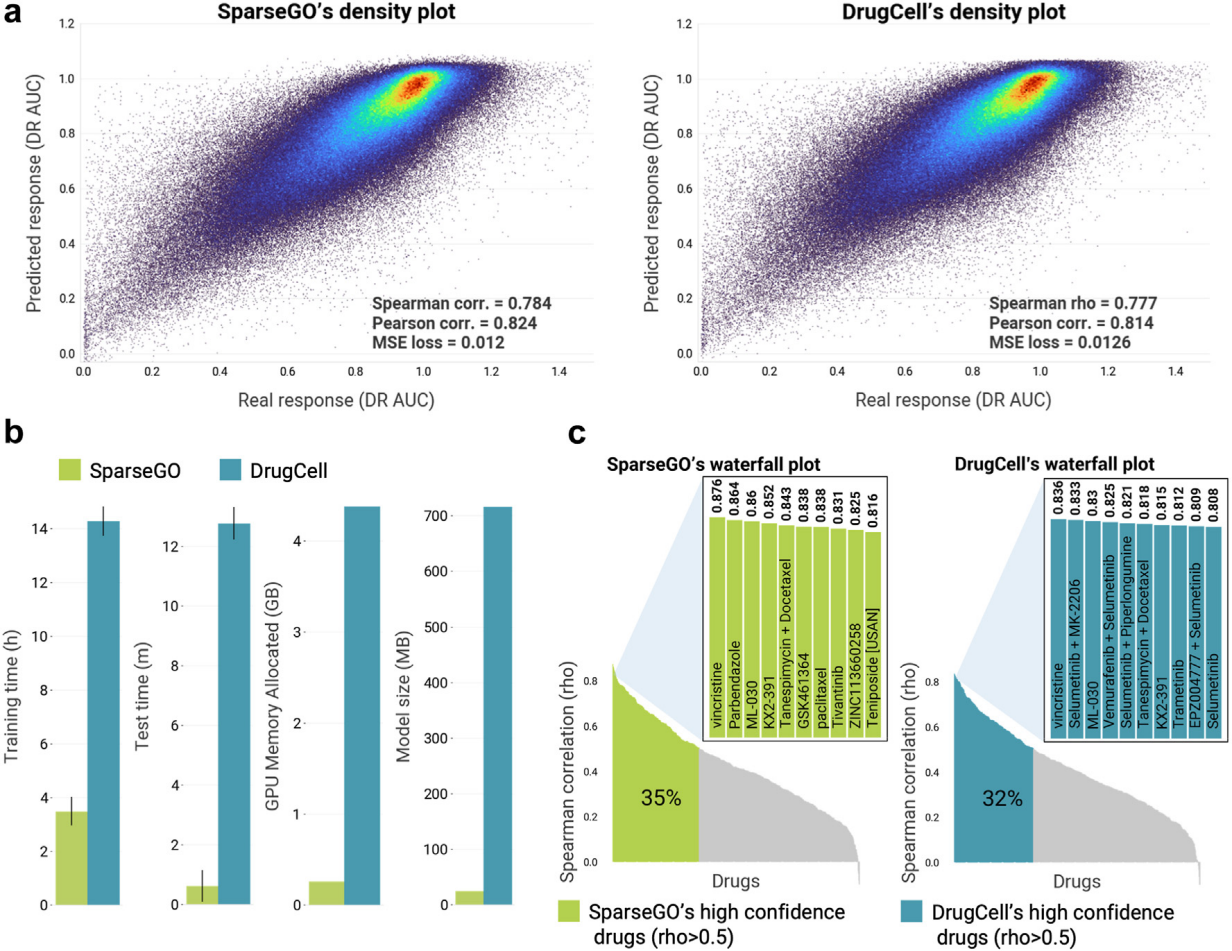
Comparison of SparseGO and DrugCell models. (a) Density plots showing predicted response (AUDRC1) versus actual response (AUDRC1) for the SparseGO and DrugCell models. Yellow/red areas represent the highest density of points. (b) Resources comparison of SparseGO versus DrugCell. (c) Waterfall plots of predicted performance of the AUDRC1 for each drug in the dataset, ranked from highest to lowest. High confidence drugs (correlation > 0.5) are highlighted in green for SparseGO and blue for DrugCell. The insets show the performance of the 10 drugs with the highest correlations.

For the sake of comparability, we first trained SparseGO with the data used to train DrugCell. We trained and cross-validated both models using very similar training conditions and by structuring them with the same GO hierarchy (3008 gene mutations and 2086 GO terms). We also used the same characteristics in the ANN branch. As shown in Fig. 6a, SparseGO’s and DrugCell’s predictions were similar: the overall accuracy of Spearman's correlation between predicted and actual AUDRC1 values increased slightly in SparseGO (DrugCell's correlation was 0.777 and SparseGO's was 0.784). These results are very similar to the one reported by the authors of DrugCell (ρ = 0.8). Since the structure of the neural network is equivalent, the difference in performance is very small. Moreover, it is particularly noteworthy that, when compared to DrugCell, SparseGO uses 80% less training time, 96% less testing time, 94% less GPU memory, and 96% less storage memory (Fig. 6b).

Then, to ascertain how many of the drugs in the dataset have high prediction accuracy (ρ > 0.5), we calculated each drug's individual performance. As shown in Fig. 6c, for DrugCell, the calculated proportion of drugs with high confidence in the predictions was 32%, and it was 35% for SparseGO. Results are, in this case, also slightly better using fewer resources. Vincristine, a common anti-microtubule agent, has the best predictive performance in both algorithms, with a correlation larger than 0.8. ML-030, KX2-391, and the combination of tanespimycin with docetaxel are also among the drugs whose effects are best predicted by both networks, highlighting once more the similarity of the models.

There is a striking difference between the overall correlation (around 0.8) and the average correlation for each drug (around 0.4) for both DrugCell and SparseGO. The meaning of this result is that, despite both methods can recall the overall effectiveness of a drug, it is much more difficult to find out the specific cell-lines that are more sensitive or resistant to each drug.

### Thorough analysis of the quality of predictions

After obtaining favourable results when comparing SparseGO with a state-of-the-art model, we proceeded to further enhance our model's generalizability. To achieve this, we trained the model using the combined dataset that incorporated the three studies and used the AUDRC2 as the target variable. In addition, taking advantage of the resource efficiency of our algorithm, we incorporated gene expression. We performed a comparative analysis between the use of mutation and expression data by training with AUDRC2, employing the three CV schemes that are adapted to different drug experimentation scenarios.

Table 4 displays the Pearson correlation coefficients between the measured and predicted AUDRC2 for both the SparseGO CV (overall correlation across the five folds) and the independent dataset test using either mutation or expression data as input.

**Table 4:** CV and independent test Pearson correlations for the different models.

Model	Type of validation	Standard	LELO	LECO
SparseGO mutations	Cross-validation	0.814	0.742	0.320
SparseGO mutations	PRISM independent test	0.713	–	0.206
SparseGO expression	Cross-validation	0.840	0.833	0.337
SparseGO expression	PRISM independent test	0.710	–	0.193

#### Standard approach

**Fig. 7:**
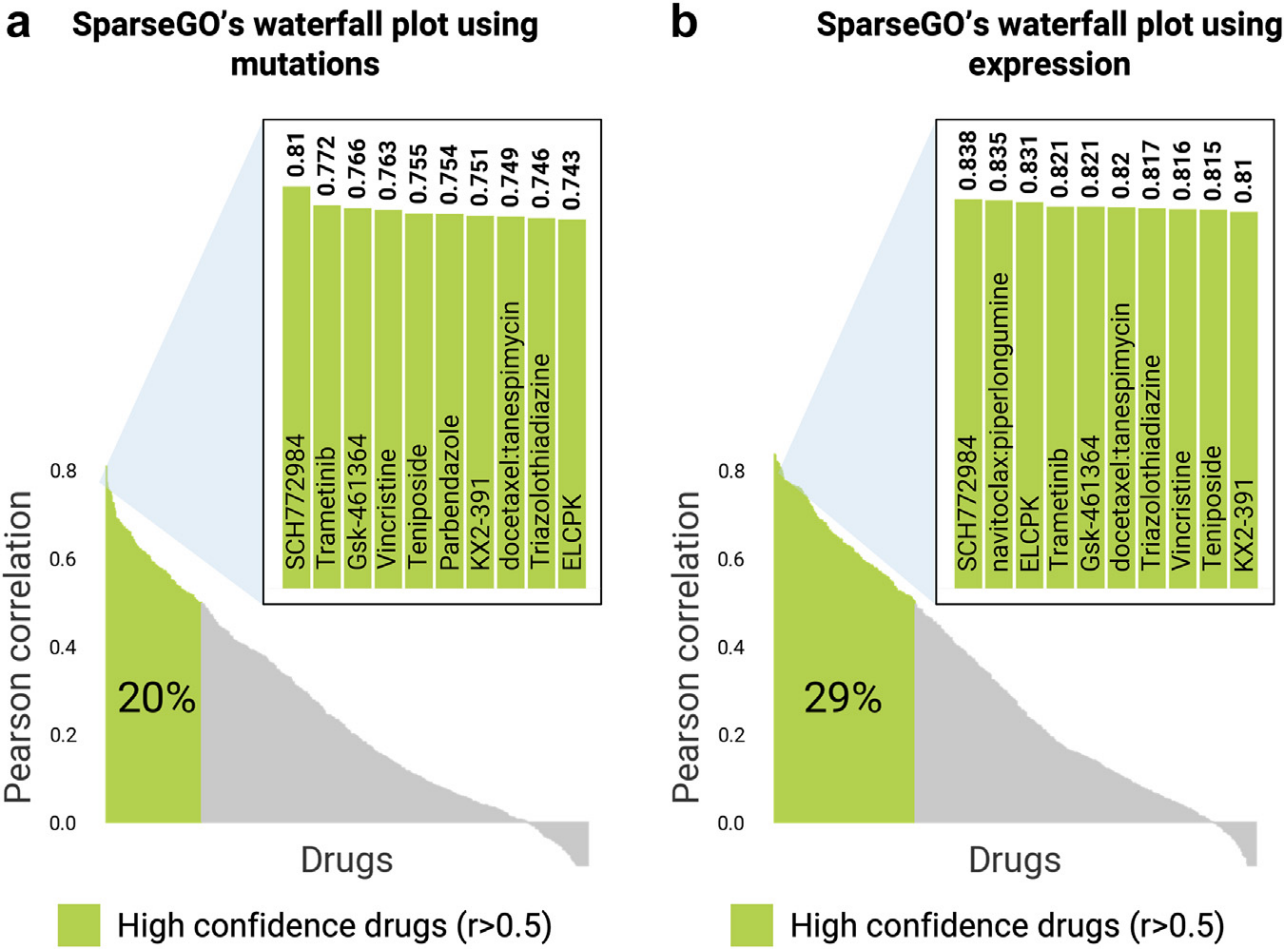
Waterfall plots of predicted performance of the AUDRC2 for each drug in the dataset, ranked from highest to lowest. High confidence drugs (correlation > 0.5) are highlighted in green. (a) Waterfall plot using the mutation model. (b) Waterfall plot using the expression model.

In the standard CV scheme, the overall Pearson correlation between the measured and predicted AUDRC2 values by SparseGO using mutations was 0.814. However, when using gene expression data, the correlation increased to 0.84. The percentage of high-confidence drug predictions was 20% when using mutations. In contrast, when predicting using gene expression, this value increased to 29% (Fig. 7). This finding is significant, as it demonstrates that the use of the expression model expands the range of more reliable drug predictions. When compared to Fig. 6c, these percentages are smaller, primarily because we used AUDRC2 instead of AUDRC1 and trained the model using three different studies, each employing diverse screening assays.

For the independent dataset test, we first calculated the Pearson correlation between the measured AUDRC2 values in PRISM and the studies that were part of the training datasets. This provided us with an approximation of the highest correlation value that could be achieved when predicting using the model. The Pearson correlations between PRISM and CTRPv2, GDSC1, and GDSC2 were found to be 0.68 (n = 98,147), 0.653 (n = 75,652), and 0.667 (n = 83,320), respectively. As presented in Table 4, the correlation between the measured and predicted AUDRC2 values by SparseGO in PRISM was 0.71, irrespective of whether mutations or gene expression data were used. This demonstrates that SparseGO constructs an ensemble model that has improved extrapolation capabilities compared to any of the previous datasets.

#### Drug-reposition approach: LELO

As evidenced by the metrics in Table 4, LELO and LECO approaches are far more challenging than the standard method. The LELO approach simulates a scenario where the model is used for drug repositioning, involving the evaluation of a drug's efficacy on an unseen cell line. When using the 3008 mutations as input, the overall Pearson correlation achieved was 0.742, which further improved to 0.833 when incorporating expression data. This outcome emphasizes the importance of integrating expression data to enhance prediction accuracy in such contexts. Notably, the performance using expression data is comparable to that of the standard CV, indicating that our model can effectively handle unseen cell lines. However, it is important to note that the genomic data used in our study was sourced from the same database (CCLE), which reduces inconsistencies and streamlines the training process.

#### In-silico drug sensitivity test approach: LECO

The LECO approach represents the most demanding scenario. When using expression data, the correlation between the measured and predicted AUDRC2 values is only 0.337. Furthermore, when testing the model on the PRISM dataset, which consists of 1144 different drugs, the correlation drops even further to 0.193. Similar results were observed in the mutations model. This outcome highlights that, at least using this approach, we are still far from achieving a comprehensive in silico simulation of the effects of drugs.

### DeepMoA: predicting the mechanism of action (MoA) using XAI

As stated, physicians need not only good predictions, but also an understanding of why. The primary goal of SparseGO is to be explainable, which is accomplished by providing accurate predictions of the response to a drug and the processes through which the drug produces its effect. After the former was demonstrated, we implemented DeepMoA, a method capable of predicting the MoA of drugs using the trained SparseGO model.

#### Assessing the XAI approach

DeepLIFT is a XAI algorithm that identifies key neurons, which in our case represent GO terms, that affect the predicted values. As shown in Fig. 5, we used DeepLIFT to compute scores for all neurons of SparseGO’s VNN branch by introducing the gene expression of all cell lines and the drugs to the trained SparseGO model. Each cell line-drug pair is given a score, which we added to maintain a global view of the importance of the neurons. At the end of the computations, each drug has 6 scores per GO term that are used as inputs to the SVMs.

**Fig. 8:**
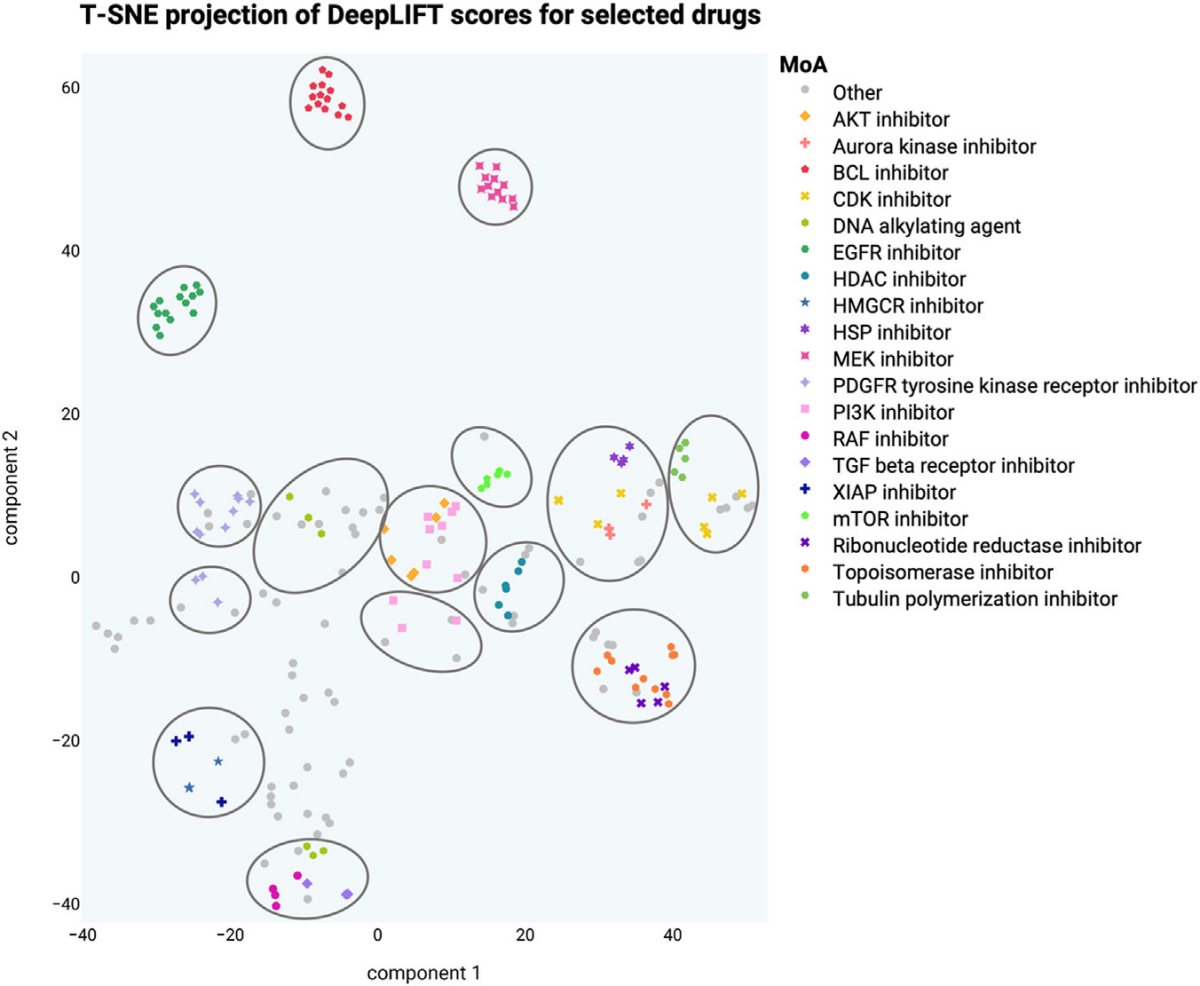
T-SNE projection of DeepLIFT scores for each drug. Each dot in the figure is a two-dimensional representation of all the DeepLIFT scores of a drug. The color of the dots symbolizes the drug's classification. The circles are clusters generated by mclust.

First, we assessed whether these scores were informative for MoA identification. Using T-SNE(25) we visualized DeepLIFT scores in a low-dimensional vector space. A known classification for some drugs was retrieved from clue.io.^29^ We selected and plotted those drugs whose classification was shared by 2 or more drugs (214 drugs). Each point in the figure represents a two-dimensional representation of all scores for a drug. We then used mclust^30^ to find clusters in that vector space. Fig. 8 shows that several compounds sharing the MoA are in the same cluster. For example, the projections of the scores between MEK inhibitors, between EGFR inhibitors and between BCL inhibitors are similar. After confirming that the DeepLIFT scores were related to the MoA, we proceeded to create the DeepMoA method.

#### DeepMoA accurately predicts the MoA of any drug

**Fig. 9:**
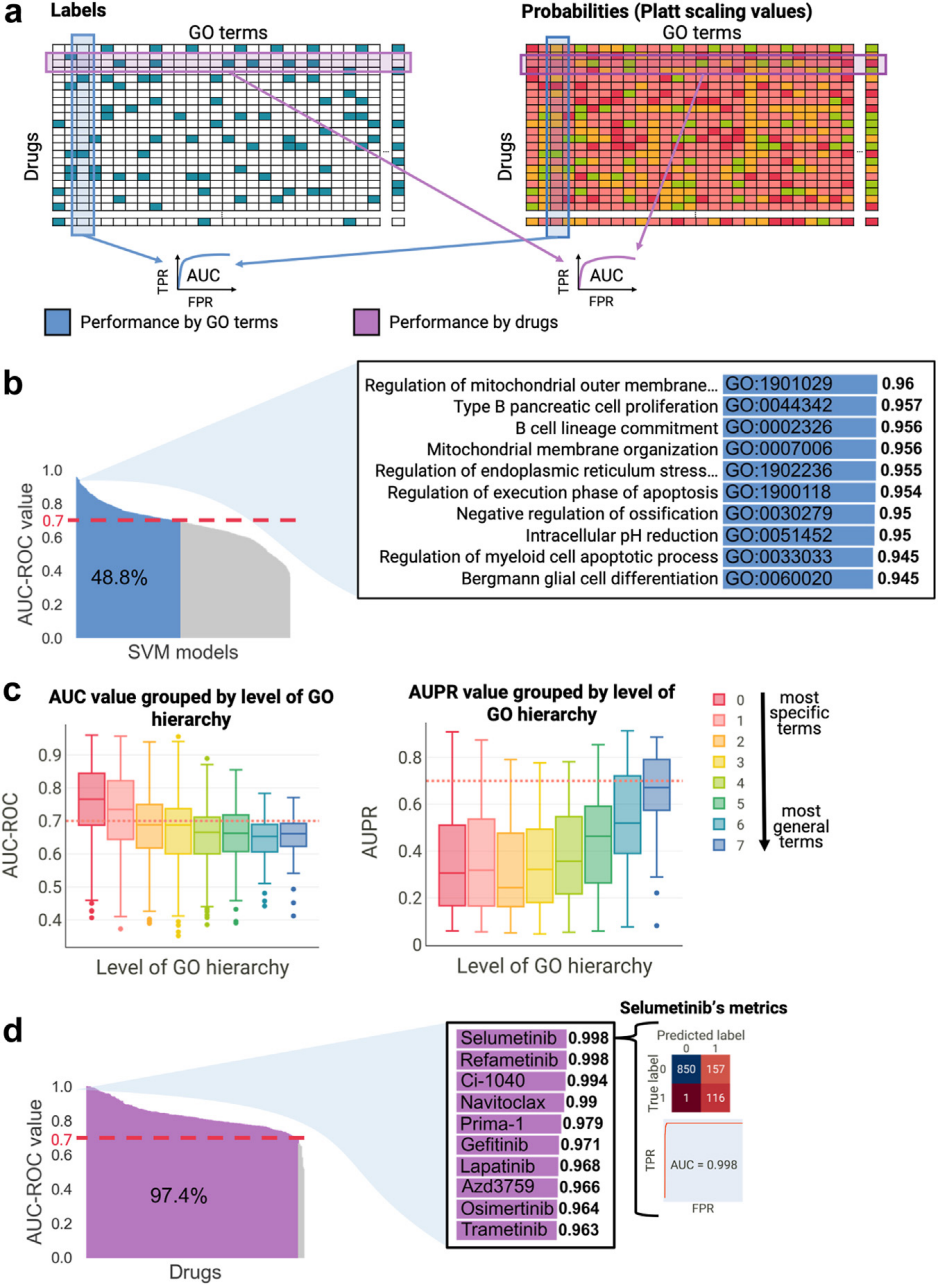
DeepMoA Validation (a) The binary matrix of labels is created using the annotations extracted from ChEMBL and CTRPv2. The probability matrix contains the Platt scaling values of the test sets resulting from the 4-fold cross-validation of the models. The performance by GO terms is measured by computing the AUROC to compare the true MoA labels of a GO term with the predicted probabilities of the same GO term. The performance by drugs is measured by computing the AUROC to compare the true MoA labels of a drug with the predicted probabilities of that drug. (b) Waterfall plot of AUROC for each SVM model (GO term), ranked from highest to lowest. Models with AUROC equal to or higher than 0.7 are highlighted in blue. The insets show the performance of the 10 models with the highest AUROC. (c) Boxplots of the AUPRs and AUROCs are shown, with GO terms grouped based on their level in the GO hierarchy. The color of the boxplot indicates the level, ranging from 0 (representing the most specific terms) to 7 (representing the most general terms). (d) Waterfall plot of AUROC for each drug, ranked from highest to lowest. Models with AUROC equal to or higher than 0.7 are highlighted in purple. The inset shows the performance of 10 drugs with high AUROC. The confusion matrix and AUROC curve of the drug with the highest AUROC is also shown.

To validate the DeepMoA method, we first determined the AUROC for each SVM model to compare the true MoA labels of a GO term with the predicted probabilities of the same GO term (Fig. 9a). We found that more than 48% of the SVM models have an AUROC higher or equal to 0.70 (Fig. 9b). As shown, regulation of mitochondrial outer membrane permeabilization involved in apoptotic signaling pathway, negative regulation of execution phase of apoptosis and intracellular pH reduction are among the best predicted MoAs. In addition, when we examined performance by GO hierarchy level (Fig. 9c), we discovered that the Area Under the Precision-Recall curve (AUPR) is bigger for general terms -i.e. it is easier to predict a general term than a specific term. However, the AUROC -where the relative size of the classes does not affect-shows that the AUROC is slightly better for more specific terms (Fig. 9c).

Then, we computed the AUROC to compare the true MoA labels with the predicted probabilities of each drug (Fig. 9a). In this case, almost all drugs had an AUROC higher than 0.7 (Fig. 9d). This suggests that when all models are used to predict for a certain drug, most of the predictions are correct. For example, Fig. 9d shows the confusion matrix obtained for a given drug (selumetinib); there are 116 true positives and 850 true negatives, versus 157 false positives and only 1 false negative. The results obtained from DeepMoA using mutations as input (shown in Supplementary Fig. S1) are not sufficiently satisfactory in terms of accuracy.

#### Findings on the top DeepMoA models

Despite the accuracy of the method being confirmed by the AUROCs, we wanted to demonstrate the method's applicability by examining in depth three interesting GO terms. We will examine three columns of the MoA matrix, i.e., we will focus on GO terms with very accurate predictions - according to cross-validation - and test whether it is possible to predict these MoAs for unlabeled drugs.

##### Regulation of ERK1 and ERK2 cascade (GO:0070372)

**Fig. 10:**
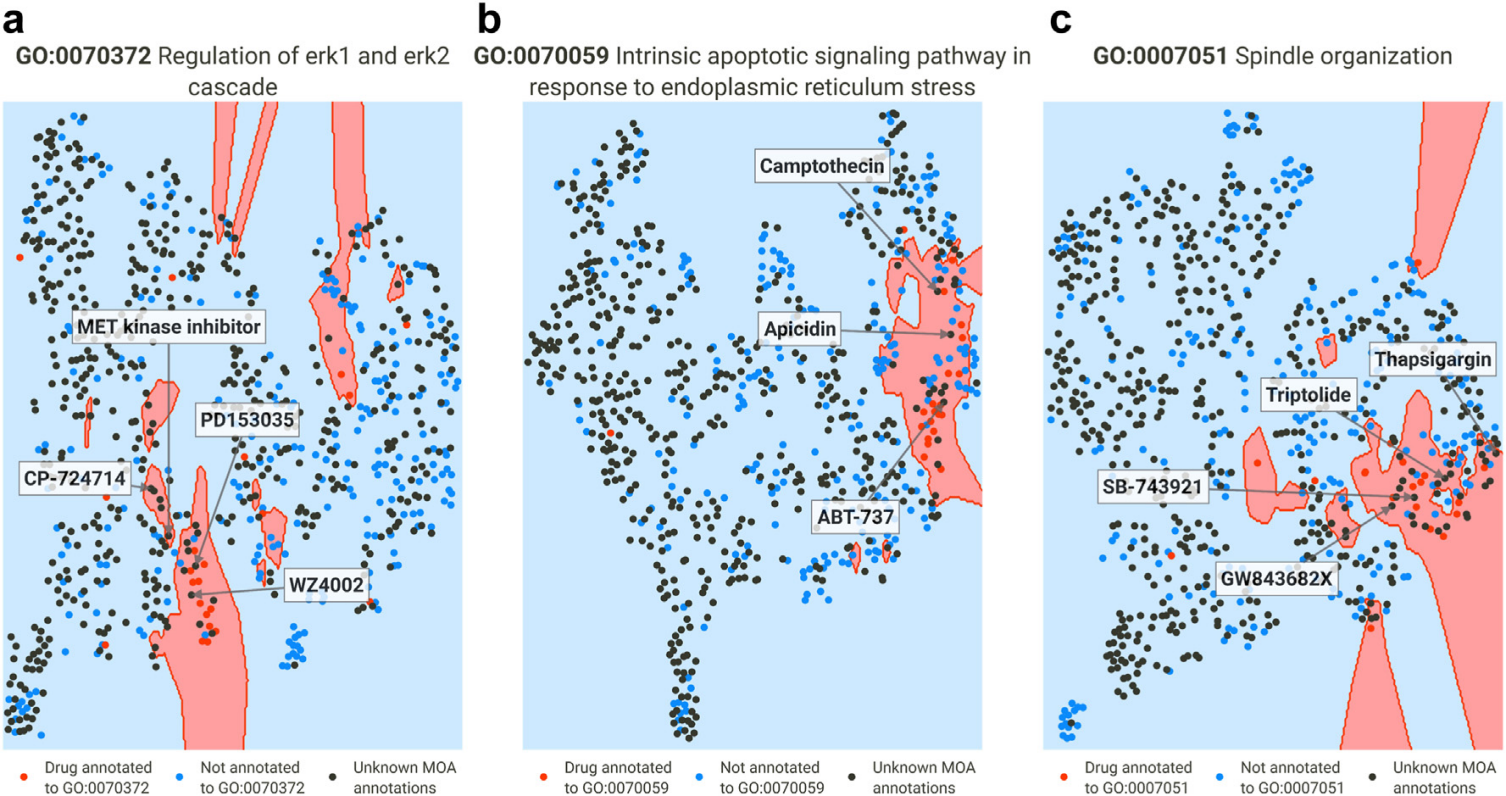
Examples of SVM models represented in two dimensions using nearest neighbors. Red dots represent drugs annotated to the term, while blue dots represent the opposite; black dots represent drugs for which no annotations were found in ChEMBL or CTRPv2 (unlabeled drugs). (a) Representation of the SVM model of regulation of ERK1 and ERK2 cascade. CP-724,714, MET kinase inhibitor, PD 153035 and WZ4002 are unlabeled drugs. (b) Representation of the SVM model of intrinsic apoptotic signaling pathway in response to endoplasmic reticulum stress. ABT-737, apidicin and camptothecin are unlabeled drugs. (c) Representation of the SVM model of spindle organization. Thapsigargin, triptolide, SB-743921 and GW84368X are unlabeled drugs.

The ERK1/ERK2 cascade, known as the MAPK pathway, plays a pivotal role in various cellular processes, including cell proliferation, differentiation, survival, and apoptosis. Dysregulation of this cascade has been implicated in the development and progression of multiple cancer types. Consequently, targeting the ERK1/ERK2 pathway has emerged as a significant strategy in cancer drug development.^31^ Among the annotated drugs, 22 of them are associated with GO:0070372. As expected, these include kinase inhibitors like EGFR and HER2 inhibitors (e.g. lapatinib, erlotinib), as well as EGFR and VEGFR2 inhibitors (e.g. vandetanib, pelitinib). Fig. 10a demonstrates the successful distinction of annotated drugs by the final SVM model (test AUROC = 0.81), while also suggesting the possibility of other unannotated drugs having the same MoA. Notably, WZ4002, an EGFR inhibitor, has been found to inhibit ERK1/2 in lung cancer^32^^,^^33^ and lung adenocarcinoma cell lines.^34^ This information taken from literature was not used during model training but it was accurately predicted to exhibit this mechanism. Similarly, literature shows CP-724714 partially inhibit ERK1/2 phosphorylation in BT-474^35^ and SKBr3 breast carcinoma cells.^36^ PD153035 has been identified as a blocker of ERK1/2 signaling in a non-oncological study.^37^ As shown in Fig. 10a, these drugs were predicted to have this MoA. Furthermore, a MET kinase inhibitor (specific name not provided) was also predicted to have this MoA. Previous studies have shown that inhibitors of this type have demonstrated efficacy in modulating the ERK1/ERK2 signaling pathway.^38^

##### Intrinsic apoptotic signaling pathway in response to endoplasmic reticulum stress (GO:0070059)

Induction of apoptosis through endoplasmic reticulum (ER) stress is a commonly observed mode of action for cancer drugs. ER stress sensors play a significant role in tumor growth, metastasis, and response to various treatment modalities such as chemotherapy, targeted therapies, and immunotherapy.^39^^,^^40^ Fig. 10b presents our developed model with a test AUROC of 0.9. Among the annotated terms, BCL-2 inhibitors like venetoclax and navitoclax, along with 21 other drugs, are included. Our predictions highlight the potential of the BCL-2 inhibitor ABT-737 to induce ER stress. This aligns with previous research demonstrating ABT-737's ability to induce ER stress in human melanoma cells^41^ and human hepatocellular carcinoma.^42^ Notably, apicidin, a fungal metabolite, has been found to induce ER stress in neuroblastoma cells^43^ and nasopharyngeal carcinoma,^44^ despite not being currently associated with the GO term. Moreover, camptothecin has been reported to trigger ER stress-mediated autophagy in human prostate cancer cells^45^; evidence for this MoA was also found in a study on Alzheimer's disease.^46^ Camptothecin was also identified as having the GO term.

##### Spindle organization (GO:0007051)

Spindle organization is a critical process for accurate chromosome segregation during cell division. Disruptions in spindle organization can lead to chromosomal instability and contribute to the heterogeneity and evolution of cancer cells.^47^ Targeting spindle-related processes is an important strategy in cancer treatment. The final SVM model (test AUROC = 0.93) identifies several drugs with known effects on spindle organization, including aurora kinase inhibitors (barasertib and ispinesib), taxanes (such as paclitaxel), and vinca alkaloids (like vincristine). Under the predicted terms we have SB-743921, which is actually a kinesin spindle protein inhibitor, and its association with spindle organization is supported by experimental evidence.^48^ For instance, studies have shown that treatment with SB-743921 leads to mitotic spindle dysfunction in cells with TP53 mutations.^49^ Furthermore, GW843682x, a polo-like kinase 1 inhibitor, has been found to disrupt spindle formation and induce abnormal mitotic processes in lung cancer cells.^50^^,^^51^ Two other drugs predicted by the model, namely thapsigargin and triptolide, have been shown to interfere with spindle activities in mouse oocytes^52^, ^53^, ^54^ (Fig. 10c).

As demonstrated, the DeepMoA method has proven to be a reliable, comprehensible and interesting approach for classifying drugs based on their MoA.

#### Examining in depth the MoA of other unlabeled drugs

Next, we will study some drugs with accurate predictions, i.e., some rows of the predicted MoA matrix. Our objective is to showcase examples of drugs whose MoA was not used during the model training process but yield predictions consistent with prior research findings. To refine the list of Gene Ontology (GO) terms and attain greater specificity, we employed a sorting method based on the logit difference. This difference represents the drug's logit score for a predicted as annotated GO term (calculated using the corresponding SVM model) minus the actual logit score for the same GO term (derived from the MoA labels matrix). Subsequently, we scrutinized the top 30 GO terms to gain insights into the predicted MoA of the drugs under investigation.

**Fig. 11:**
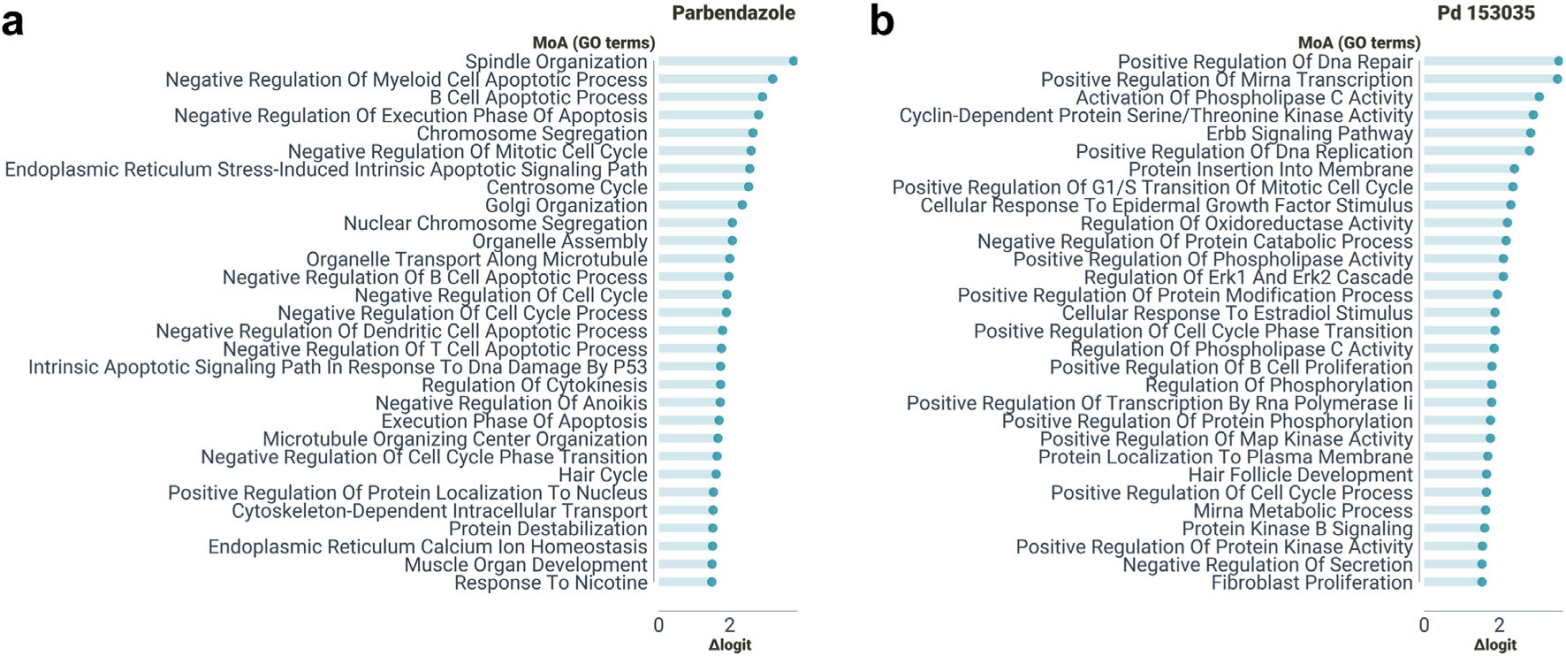
MoA predictions examples. GO terms are ordered by logit differences, only the 30 GO terms with the largest difference are shown. (a) Parbendazole’s top predictions. (b) PD153035’s top predictions.

Parbendazole, a potent inhibitor of microtubule assembly, has demonstrated its ability to depolymerize cytoplasmic microtubules, resulting in the presence of only one or two microtubules associated with a centriole.^55^^,^^56^ This disruption significantly impairs the formation and function of the mitotic spindle, leading to notable defects in chromosome segregation and organization.^57^ Our method, as depicted in Fig. 11a, provides compelling evidence of the close association between parbendazole treatment and these critical biological processes. Specifically, our predictions indicate a strong relation with terms such as chromosome segregation (GO:0007059), nuclear chromosome segregation (GO:0098813), centrosome cycle (GO:0007098), negative regulation of mitotic cell cycle (GO:0045930), and notably, spindle organization (GO:0007051), organelle transport along microtubule (GO:0072384) and microtubule organizing center organization (GO:0031023). In the context of pancreatic cancer (PC), a study revealed that parbendazole, besides affecting microtubule organization, inhibits cell cycle progression by inducing G2/M arrest, promoting apoptosis, and causing DNA damage. Furthermore, it was discovered that p53 status in cells treated with this drug influenced the appearance of polyploid cells.^58^ Our predictions support these findings by suggesting the involvement of parbendazole in different cell cycle and apoptosis-related terms and in the negative regulation of intrinsic apoptotic signaling pathway in response to DNA damage by p53 class mediator (GO:1902166). Furthermore, recent research on mitochondrial toxicity demonstrated that parbendazole-treated cells display nuclear alterations, including endoplasmic reticulum vacuolization, mitochondrial redistribution, and cytoskeleton destabilization.^59^ These processes are associated with Golgi organization (GO:0007030), organelle assembly (GO:0070925), cytoskeleton-dependent intracellular transport (GO:0030705) and endoplasmic reticulum calcium ion homeostasis (GO:0032469), which were also predicted by DeepMoA.

PD153035 is a tyrosine kinase inhibitor known for its specific and potent inhibition of the epidermal growth factor receptor (EGFR) tyrosine kinase, which is part of the ErbB receptor family.^60^ Our predictions, as shown in Fig. 11b, support this definition by including terms such as ErbB signaling pathway (GO:0038127) and cellular response to epidermal growth factor stimulus (GO:0071364). In cervical cancer experiments, PD153035 treatment led to a significant suppression of EGFR expression, as well as the phosphorylation of PI3K and AKT.^61^ Results indicate the relationship of the drug with terms associated to protein serine/threonine kinases and protein kinase B. In chronic myeloid leukemia studies, altered expression levels of key signaling molecules in the EGFR/MAPK pathway were observed in PD153035-treated mouse groups.^62^ As stated before, PD153035 was found to block ERK1/2 signaling.^37^ DeepMoA predictions include positive regulation of MAP kinase activity (GO:0043406) and Regulation of ERK1 and ERK2 cascade (GO:0070372). Interestingly, predictions also includes a term associated with hair: hair follicle development (GO:0001942). It is known that EGFR signaling plays a role in the normal hair cycle, and inhibiting EGFR signaling can disrupt the progression from the anagen to telogen phase, leading to the formation of disorganized hair follicles.^63^

In addition to the study of GO terms, we applied DeepLIFT to the input layer of cell lines. By examining the value of the resulting DeepLIFT scores, it is possible to identify the genes that contribute to predicting the response of each drug. Supplementary Fig. S2 shows a heat map of the 50 genes with the highest variance for the drugs shown in Fig. 8 in the case of the mutation study. In the Supplementary material we also include an Excel file with the attribution score for each gene/cell line pair when using mutations as input, as well as two Excel files (one using mutations as input and one using expression) with the 50 genes with the highest absolute value of attribution for each treatment.

## Discussion

We have described SparseGO, a neural network designed to predict drug efficacy in cancer cell lines. SparseGO uses a VNN architecture incorporating the Gene Ontology hierarchy in order to address the interpretability challenges often associated with machine learning approaches.

We conducted an extensive comparison between SparseGO and DrugCell, a state-of-the-art model with a similar structure. Notably, our approach demonstrated significantly improved efficiency in the use of computing resources by using sparse matrices. Specifically, SparseGO required 94% less GPU memory, 80% less training time, and 96% less testing time compared to DrugCell under very similar training conditions. This finding highlights the advantage of employing sparse matrix layers to represent ontologies, as opposed to meticulously connecting each neuron to its corresponding parents. This efficient resource utilization enabled us to extend the model's capabilities by incorporating gene expression data. In contrast, the DrugCell model exhausted GPU memory and could not be tested with gene expression data. Since DrugCell and SparseGO (using mutations as input) have a similar architecture, their results are also similar. Any minor differences observed in favour of SparseGO may be attributed to the different parameter tuning approaches employed.

In the context of genomic and response profiling studies, the presence of inconsistencies arising from variations in data acquisition poses a significant challenge. As studied in,^18^ this challenge highlights the likelihood that machine learning models trained on a specific data source may encounter difficulties when applied to alternative datasets. To confront this issue head-on and foster the development of more reliable and generalized models, after comparing our model to the state-of-art-model, we adopted a multifaceted approach. By harnessing a dataset encompassing information from three distinct studies (CTRPv2, GDSC1 and GDSC2), we curated a comprehensive and diverse training dataset using a comparable drug-response metric. Additionally, we evaluated the model’s performance on an independent dataset (PRISM) to test its effectiveness on previously unseen data. Furthermore, we incorporated different CV schemes to test the model on different scenarios.

In addition to the standard K-fold CV, we incorporated two additional CV schemes. The LELO approach was specifically designed for drug repositioning scenarios, where cell lines that have not been previously tested with any drug are involved. On the other hand, LECO approach was implemented for in silico sensitivity analysis, where unseen drug's effectiveness is estimated. Our findings demonstrate that these two scenarios, particularly the latter, pose greater challenges compared to the initial approach. Despite their demanding nature, the predictions achieved using gene expression as input in the LELO approach remained accurate, with a correlation of approximately 0.83 between the predicted and real AUDRC2. The observed results can be in part attributed to the utilization of genomic data from a single source. To improve the model's robustness, it is important to consider training models using more diverse data in future studies.

In the LECO cross-validation scheme, results were far from perfect. Previous studies, such as the work conducted by Zagidullin et al., have compared various methods for parameterizing a drug's structure. Among these methods, the Morgan fingerprint, which was used in our study, is considered one of the best “standard” approaches.^64^ However, the research suggests that alternative drug parameterization techniques, such as convolutional autoencoders, exhibit superior performance compared to the Morgan fingerprint. The adoption of these alternative methods holds promise for enhancing the quality of predictions, particularly within the LECO cross-validation scheme. Incorporating autoencoder-generated data represents an intriguing avenue for future exploration, offering potential improvements in prediction accuracy.

The primary objective of this study is to address the challenge of interpretability in ML by not only predicting drug response but also providing explanations for the predictions. To achieve this, we used an XAI algorithm to explore the network structure and establish a ground truth for the MoA of a subset of drugs.

DeepLIFT assigns an attribution score to each neuron in the network. DeepLIFT represents a fast and innovative extension of sensitivity-based methods since it can address saturation issues that may arise in other perturbation-based and gradient-based approaches. To establish the ground truth for MoA of the drugs in our study, we adopted a simple approach. For drugs with a known protein target, we considered all the GO terms associated with that target as potential MoAs for the drug. Additionally, if the CTRPv2 dataset provided specific GO terms related to the MoA of certain drugs, we included those terms and their parent terms within the ontology.

We confirmed the accuracy of DeepMoA by measuring the performance using the AUROC by GO terms and by drugs. Then, by analysing the biological processes: GO:0070372, GO:0070059, and GO:0007051, we showed its applicability. The method correctly classified some unlabeled drugs. Finally, by predicting and studying parbendazole and PD153035, we exemplified how the method can be used and, again, its accuracy. While wet lab experiments were not within the scope of our current work, we acknowledge the need for experimental validation to validate predictions. In future studies, conducting wet lab experiments would be valuable, particularly for drugs without established state-of-the-art MoA or those that may involve unstudied effects.

Although nothing impedes testing DeepLIFT on the characteristics of the molecule, the challenges in interpreting the individual elements of the Morgan fingerprint and their relationship to specific substructures of the molecule, prevents us computing the DeepLIFT score for that particular part of the input. Exploring the application of XAI to extract information of compounds, such as pharmacophore fragments, based on network output is a promising direction for future research. If the fingerprint were to incorporate substructure patterns of the drugs, the DeepLIFT algorithm could potentially uncover the key structures responsible for the drug's effectiveness. Additionally, leveraging ontologies like ChEBI,^65^ which contain annotated compounds, may facilitate the identification of fragments within the molecule that exhibit high attribution scores (using the DeepLIFT method). These ideas could lead to a more comprehensive understanding of the MoA of drugs.

SparseGO narrows the gap between developers and clinicians. It offers flexibility in selecting the input data that best suits their needs, ensuring adaptability and applicability in diverse scenarios. The training and testing process of the model is designed to accommodate the availability of easily accessible computational resources. Moreover, SparseGO takes into consideration the inherent challenges associated with generalization, enhancing its ability to provide reliable predictions. Additionally, by using DeepMoA, the network not only offers drug alternatives but also uncovers the underlying factors driving these predictions, empowering clinicians with deeper insights and decision-making support.

## Contributors

AR conceived this study. AR and KS verified the underlying data. AR and KS conceptualized and developed the algorithms. KS carried out the computational implementation. Both authors actively contributed to drafting, reviewing, and approving the manuscript for submission.

## Data sharing statement

The codebase, DeepMoA method, trained models, and training data for SparseGO can be accessed and downloaded from the GitHub repository at https://github.com/KatynaSada/SparseGO.

Raw data from screening experiments for the four datasets (CTRPv2, GDSC1, GDSC2, and PRISM) can be obtained through the PharmacoGX platform.

The pairs used to train the mutation models are available for retrieval on DrugCell's web portal at http://drugcell.ucsd.edu/downloads.

The CCLE expression data used in this study, specifically the 22Q2 dataset, can be downloaded from the depmap portal (https://depmap.org/portal/download/all/).

These resources and datasets are made available to facilitate further research and support reproducibility.

## Declaration of interests

The authors declare that they have no competing interests.

## Appendix ASupplementary data

Supplementary Figs. S1 and S2

Attribution data all genes_mut

Top genes_exp

Top genes_mut
