# Supplementary material for: Discovering the mechanism of action of drugs with a sparse explainable network
Source: eBioMedicine. 2023 Aug 24;95:104767. doi: 10.1016/j.ebiom.2023.104767 (PMC10474372; doi:10.1016/j.ebiom.2023.104767)
Supplement: Supplementary Figs. S1 and S2 [file mmc1.docx]

Supplementary: Discovering the Mechanism of Action of Drugs with a Sparse Explainable Network

Katyna Sada Del Real^1^, Angel Rubio^1,2*^

^1^Departamento de Ingeniería Biomédica y Ciencias, TECNUN, Universidad de Navarra, San Sebastián, 20018, Spain.

^2^Instituto de Ciencia de Datos e Inteligencia Artificial (DATAI), Universidad de Navarra, Pamplona, 31080, Spain

* To whom correspondence should be addressed. Email: arubio@tecnun.es

# Supplementary figures


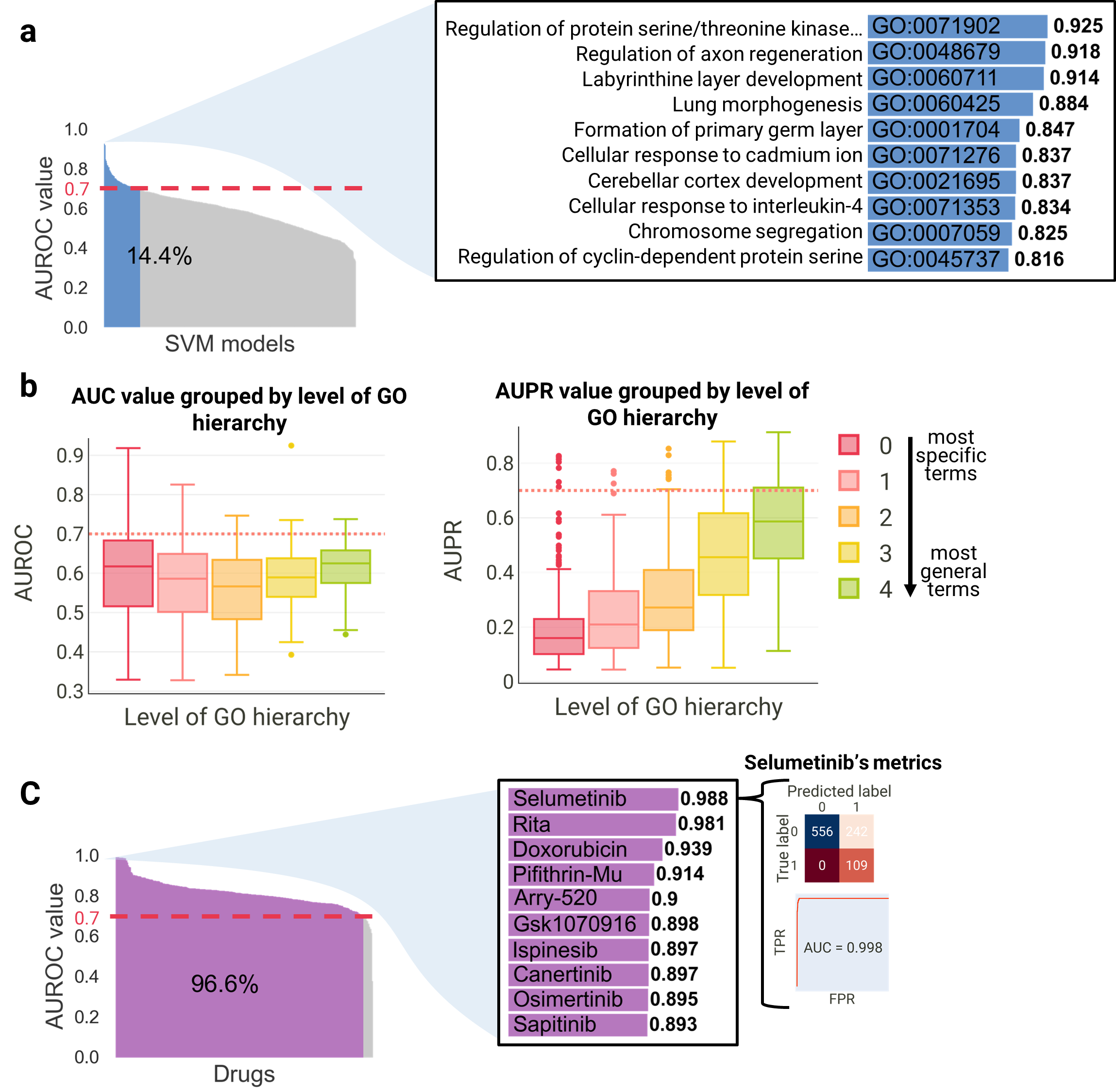


Supplementary Figure S1 Results of DeepMoA method using the mutation model (a) Waterfall plot of AUROC for each SVM model (GO term), ranked from highest to lowest. Models with AUROC equal to or higher than 0.7 are highlighted in blue. The insets show the performance of the 10 models with the highest AUROC. (b) Boxplots of the AUPRs and AUROCs are shown, with GO terms grouped based on their level in the GO hierarchy. The color of the boxplot indicates the level, ranging from 0 (representing the most specific terms) to 7 (representing the most general terms). (c) Waterfall plot of AUROC for each drug, ranked from highest to lowest. Models with AUROC equal to or higher than 0.7 are highlighted in purple. The inset shows the performance of 10 drugs with high AUROC. The confusion matrix and AUROC curve of the drug with the highest AUROC is also shown.


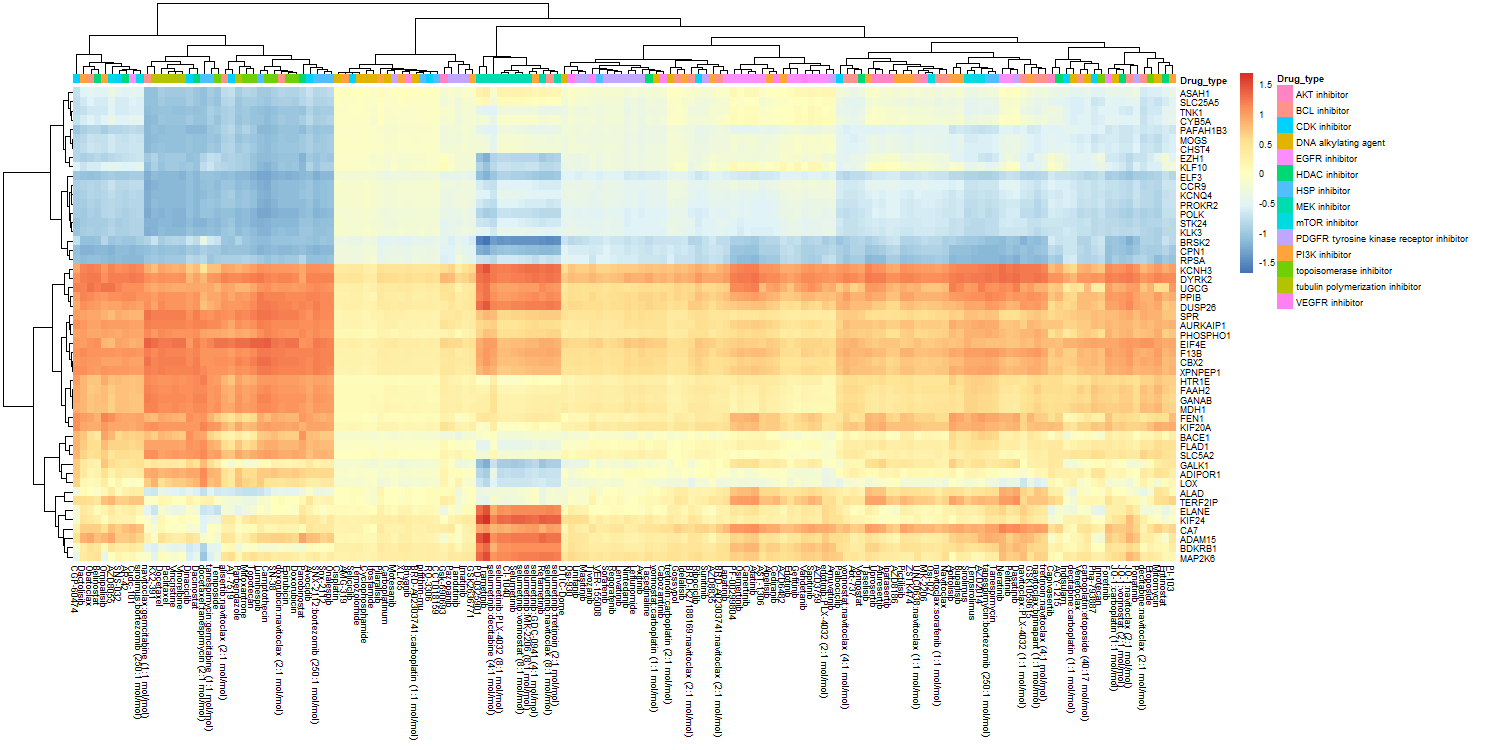


Figure S2 Heat map of the 50 genes with the highest variance for the drugs shown in Figure 8 in the case of the mutation study.
